# Cost-effectiveness of an ambulance-based referral system for emergency obstetrical and neonatal care in rural Ethiopia

**DOI:** 10.1186/s12884-017-1403-8

**Published:** 2017-07-12

**Authors:** Sandro Accorsi, Edgardo Somigliana, Hagos Solomon, Tsegaye Ademe, Jofrey Woldegebriel, Biadgo Almaz, Mohammed Zemedu, Fabio Manenti, Akalu Tibebe, Pasquale Farese, Aberra Seifu, Serena Menozzi, Giovanni Putoto

**Affiliations:** 1 Project “Italian Contribution to the Health Sector Development Programme and Contribution to the MDG Fund”, Addis Ababa, Ethiopia; 2 Fondazione Ca’ Granda, Ospedale Maggiore Policlinico and Università degli Studi di Milano, Milan, Italy; 3 Doctors with Africa CUAMM, Addis Ababa, Ethiopia; 4 St. Luke Catholic Hospital and College of Nursing & Midwifery, Wolisso District, South West Shoa, Oromia region, Ethiopia; 5 Doctors with Africa CUAMM, Padua, Italy; 6 Health Department of South West Shoa Zone, Oromia region, Ethiopia; 7 Dept Obstet-Gynecol, Fondazione Ca’ Granda, Ospedale Maggiore Policlinico, Via Commenda, 12 20122 Milan, Italy

**Keywords:** Ambulance, EmONC, Remote setting, Cost-effectiveness

## Abstract

**Background:**

To estimate the cost-effectiveness of an ambulance-based referral system an dedicated to emergency obstetrics and neonatal care (EmONC) in remote sub-Saharan settings.

**Methods:**

In this prospective study performed in Oromiya Region (Ethiopia), all obstetrical cases referred to the hospital with the ambulance were consecutively evaluated during a three-months period. The health professionals who managed the referred cases were requested to identify those that could be considered as undoubtedly effective. Pre and post-referral costs included those required to run the ambulance service and the additional costs necessary for the assistance in the hospital. Local life expectancy tables were used to calculate the number of year saved.

**Results:**

A total of 111 ambulance referrals were recorded. The ambulance was undoubtedly effective for 9 women and 4 newborns, corresponding to 336 years saved. The total cost of the intervention was 8299 US dollars. The cost per year life saved was 24.7 US dollars which is below the benchmarks of 150 and 30 US dollars that define attractive and very attractive interventions. Sensitivity analyses on the rate of effective referrals, on the costs of the ambulance and on the discount rate confirmed the robustness of the result.

**Conclusions:**

An ambulance-based referral system for EmONC in remote sub-Saharan areas appears highly cost-effective.

## Background

Maternal and neonatal mortality remain an unsolved health priority in low income countries and sub-Saharan Africa in particular [1]. Most maternal deaths are actually preventable and occur during labour, delivery and the first day postpartum. Skilled attendance at birth is the most important intervention to reduce maternal and neonatal mortality since complications leading to these deaths are unpredictable but can be successfully treated if diagnosed early and properly managed [2–4]. Therefore, the strategy has shifted in the last decade from the risk approach, involving identification of high risk pregnancies which can develop complications, to provision of skilled care during delivery and Emergency Obstetric and Neonatal Care (EmONC) when a complication occurs. An integrated and comprehensive health program is recommended to address the three delays hampering access to safe motherhood services: (i) seeking appropriate medical care for an obstetric emergency; (ii) reaching an appropriate EmONC facility; and (iii) receiving adequate care when the facility is reached [5]. However, efforts to improve access to EmONC mainly focused on addressing harmful traditional beliefs and practices, poor infrastructure and inadequate care at health facilities, while overcoming transport barriers is a relatively neglected area [1]. Albeit scanty, evidence from the few available studies emerging from disparate locations is however highly promising [6–10]. For example, in a rural area of Burundi, the integration of ambulance network with EmONC referral systems was estimated to reduce maternal mortality by 74% [8].

Overall, the performance of ambulance services for EmONC and their cost-effectiveness remain largely unevaluated in sub-Saharan settings and further evidence is warranted prior to definitely recommend its systematic implementation. To add information on this issue, we thus designed a prospective observational study in the rural area of Oromiya Region (Ethiopia) aimed at estimating the cost-effectiveness of an ambulance-based referral system specifically dedicated to EmONC.

## Methods

### Setting

Ethiopia counts around 90 million inhabitants, with over 80% of the population living in rural areas and remains one of Africa’s poorest countries [11, 12]. The percentage of deliveries attended by skilled health personnel and the maternal mortality ratio are estimated at 14% and 676 per 100,000 live births, respectively [13, 14]. The annual Gross Domestic Product (GDP) is 550 US dollars per capita, while the health expenditure per capita is 21 US dollars [15].

Southwest Shoa Zone (Oromiya Region) has 12 districts with a total population of 1,079,814 people. The percentage of institutional deliveries was estimated at 48%. The study was conducted in four of these districts (with a total population of 397,573): Wolisso Urban (50,657), Wolisso Rural (178,633), Goro (55,195) and Wonchi (113,088). The expected deliveries per year was 13,518 and the expected number of caesarean sections was 176 (local rate of 2.7%) [10]. In the four districts, there are one hospital, 18 Health Centres and 90 Health Posts. The referral facility is the St. Luke Hospital located in Wolisso town, capital of the Southwest Shoa Zone; it is a private, non-profit hospital belonging to the Ethiopian Catholic Bishop Conference.

The area is supported by a public health programme partly financed by the Italian Development Cooperation and locally coordinated by the Italian Non-Governmental Organization (NGO) *Doctors with Africa – CUAMM*. General support (personnel, rehabilitations and constructions, training, supervisions, equipments and drug supply) are given to the 21 Health posts, 8 Health Centers and the hospital that actually provides free maternal and child services.

Free ambulance services are provided to transport labouring mothers from village to nearby Health Centers and, if needed, to St. Luke Hospital. Referral directly from the Health Centers could also be done. The main vision of the intervention was to direct uncomplicated deliveries to the Health Centres and complicated cases to the hospital [10, 16]. The ambulance service was ensured with a single vehicle for 24 h a day and managed by two drivers. The drivers did not receive specific training for health assistance. However, they were instructed to inquire about the severity of the situation with the healthcare professionals of the hospital or the Health Centers. No healthcare professionals accompanied the driver (even if in some particularly critical cases, personnel of the hospital or the Health Centers could do it). The ambulance was equipped with a single couch but could carry up to three other seated persons if needed. The service was dedicated to pregnant women but, in selected cases, other urgent cases could be referred (but the potential benefits of these referrals were not included in the present analysis).

The call for the ambulance service was usually made by the pregnant woman or her family member through cell phone to the ambulance drivers’ cell phone or to a fixed phone located at the Hospital. The ambulance was generally located in the hospital. The distances between the hospital and the Health Centers varied between 2.5 and 47 Kilometres. All connecting roads in the area were rough. Only one ambulance was bought and no replacing car was available. In case of breakdown or car maintenance, the service was temporarily suspended.

### Design

The study prospectively examined the ambulance call-outs and transfers to St. Luke Hospital of women at risk of or with obstetric complication from 7th of January 2015 to 15th of April 2015 (with a one-week interruption for ambulance maintenance). Ethical approval was obtained from the Southwest Shoa Zonal Health Department. Only verbal informed consent to participate was obtained because the majority of treated women were illiterate.

The study design is reported in details in a previous publication of our group [7]. Briefly, all obstetrical cases referred to the hospital by ambulance were evaluated and managed by two health professionals with specific skills on EmONC. In particular, they were requested to independently judge the effectiveness of referral by classifying cases into three categories: *not effective*, p*ossibly effective* and *undoubtedly effective.* Initial location and distance from the hospital (and thus the relevance of the potential delay in referral with other means) was also taken into consideration in the evaluation. In particular, referrals were considered u*ndoubtedly effective* when they were thought to save the maternal and/or the neonatal life and when the use of other means to refer would have not. Judgments were given separately for the mother and the newborn and had to be given within 24–48 h of the event. The effectiveness categories were predefined but the definition was not stringent, allowing the adaptation of the decision to the specific clinical conditions and distance from the hospital. When the two health professionals disagreed on the effectiveness judgment, a third health professional was involved to make a final decision.

### Cost-effectiveness analyses

The analysis was carried out from the perspective of the District Health provider. The ultimate aim of the paper was to provide a tool to help health authorities operating in remote setting make rational choices. Even if, in the particular setting of the present study, important financial support came from external stakeholders and the hospital was private, the analysis was done assuming that, in other contexts, all costs would have to be supported by the District Health provider. Moreover, we excluded the additional costs that are initially required for starting the service (training, supervision, advertisement, health education and advocacy) because we aimed at evaluating the cost-effectiveness of the system in everyday clinical practice, not in the starting phase.

Pre and post-referral costs falling on the hospital with regards to the ambulance referrals were estimated as previously reported in details [7]. Specifically, we included costs associated with the referral system, i.e. all the costs required for running the ambulance service as well as those required to assist the woman once she reached the hospital. The main vision was that women, if not referred with the ambulance, would have not received assistance in the hospital at all. We thus included only costs that were different from those related to the assistance in the Health Centers (i.e. caesarean section, uterine evacuation, second-line uterotonic agents, fluids and parental antibiotics). Personnel costs in the hospital were excluded since 24-h assistance was already available prior to the implementation of the project with no additional need to increase the number of duty personnel after the implementation of the ambulance service. Moreover, financial support given for the improvement of the hospital and the Health Centres were excluded since these were not intended to specifically support the ambulance service. Costs of the specific medical services given to the referred women were estimated based on average cost of each medical procedure set at St. Luke Hospital. For example, the mean cost for caesarean section was estimated at 65 US dollars.

The benefits were estimated on the number of years saved based on the local life-expectancy tables [17]. Prevention of disabilities was not included in the model. A 3% discount of the life years gained was used [18]. The main analysis focused on “undoubtedly effective” referrals but we performed a secondary analysis including also cases classified as “possibly effective”. Sensitivity analyses were carried out for the costs of the ambulance, for the proportion of undoubtedly effective cases and increasing the discount of the life years gained to 6%. The intervention was deemed acceptable if the costs per each year saved was below the GDP per person per year in the country (550 US dollars), attractive if <150 US dollars and very attractive if <30 US dollars [19].

## Results

A total of 111 ambulance referrals to St. Luke Hospital were recorded during the study period. Six (5%) were from the village via Health Center to the hospital while the remaining 105 (95%) were referred from the Health Center to the hospital. All 18 Health Centers referred at least one woman. The highest number of cases (*n* = 30) came from Chitu, a Health Center located at 9 km from the hospital. Most women were aged 20–34 years (*n* = 81, 73%). Forty-six women (41%) were nulliparous while 24 (22%) had four or more previous deliveries. At the hospital level, 41 (37%) mothers were diagnosed with one or more pregnancy related complications. One maternal death and nine stillborns were recorded.

The referrals were considered undoubtedly and possibly effective for the mother and/or the newborn in 9 (8%) and 27 (24%) cases, respectively (total of 36 cases, corresponding to 32%). The ambulance was undoubtedly effective for 9 women and 4 newborns. It was possibly effective for an additional 22 women and 23 newborns. Details are shown in Table 1. The main diagnoses of the remaining 75 non-effective referrals were as follows: normal labour with vaginal delivery (*n* = 52), obstructed labour with vaginal delivery (*n* = 11), spontaneous abortion with minimal bleeding (*n* = 10), abdominal trauma without consequences (*n* = 1) and death at arrival (*n* = 1).

**Table 1:** Clinical findings of the cases judged as undoubtedly or possibly effective referrals

Clinical condition	Number of women	Number of newborns
Undoubtedly effective		
Incomplete abortion (immediate blood transfusion)	2	0
Uterine rupture, foetus alive	1	1
Impending uterine rupture, foetus alive	2	2
Uterine rupture, foetus dead	2	0
Eclampsia, foetus dead	1	0
Postpartum haemorrhage, foetal distress	1	1
Total	9	4
Possibly effective		
Preeclampsia, fetus dead	1	0
Instrumental delivery	3	3
Obstructed labour, immediate caesarean section	4	5^a^
Obstructed labour, no caesarean section	2	3^a^
Incomplete abortion	2	0
Foetal distress	0	2
Twin delivery	0	2^a^
Premature rupture of membranes, fetus alive	0	2
Premature rupture of membranes, fetus dead	1	0
Prolonged labour	3	3
Previous caesarean section in labour	1	1
Malpresentation/Transverse lie, fetus alive	2	2
Malpresentation/Transverse lie, fetus dead	3	0
Total	22	23

^a^Twin delivery (one woman corresponds to two newborns)

The extrapolated cost of the ambulance referral system for the three months period, based on the cost of one entire year (2014) is displayed on Table 2. In the three months period, the total cost of the ambulance was 6587 US dollars and the additional cost incurred to the hospital for the provision of effective referral services was 1712 US dollars which gives a total cost of 8299 US dollars. Considering only undoubtedly effective referrals, the total years saved was estimated at 336. The cost per year life saved was thus 24.7 US dollars which is below the three benchmarks (550, 150 and 30 US dollars) and fulfils the criterion to be defined as very attractive (< 30 US dollars).

**Table 2:** Costs of the ambulance

Expenses	Costs per unit	Extrapolated costs per year	Costs for the study period (3 months)
Car (Toyota Land Cruiser)^a^	44,635	11,159	2790
Car insurance per year	591	591	148
Referral system			
Mobile phones^b^	125	31	8
SIM cards^b^	14	4	1
Air-time	180	180	45
Fuel	4653	4653	1163
Car maintenance^c^			
Service	928	928	232
Damages Repair	2588	2588	647
Tyre Repair – substitution	2375	2375	594
Drivers gross salaries (*n* = 2)	3759	3759	940
Drivers’ uniform cloth	75	75	19
Total	59,923	26,343	6587

Costs are expressed in US dollars, with an exchange rate of 1 USD = 20 Ethiopian Birr

^a^The ambulance is considered to serve for 4 years

^b^Mobile phones and Sim cards were provided for the drivers. The mobile apparatus is estimated to serve for 4 years

^c^In order to temper possible variations of the costs of maintenance, it was calculated as 25% (3 out of 12 months) of the total expenses recorded over the last year of use

**Fig. 1:**
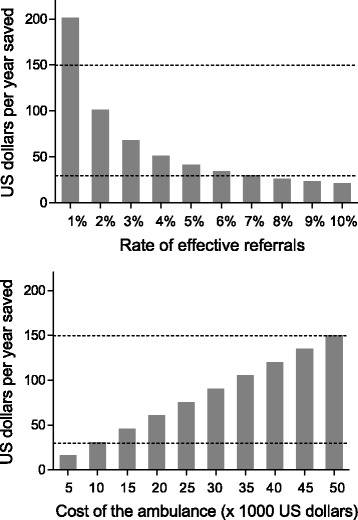
Sensitivity analyses according to the proportion of undoutedly effective referrals (upper panel) and the cost of the ambulance (lower panel). In the two panels, the two dotted lines represent the benchmarks to define attractive interventions (150 US dollars, upper line) and very attractive interventions (30 US dollars, lower line). The costs of the ambulance refer to a three months period of use

Sensitivity analyses were firstly carried out for the costs of the ambulance referral system and the rate of undoubtedly effective referral (Fig. 1). The intervention remains attractive (< 150 US dollars per year saved) up to a rate of undoubtedly effective cases of 1.3% and up to a three-months cost of the ambulance of 50,400 US dollars. Increasing the discount of the life years gained from 3% to 6% lead to a cost per year life saved of 40.5 US dollars, thus above the threshold of very attractive interventions (30 US dollars) but below the threshold for attractive interventions (150 US dollars). Finally, all the analyses were repeated considering together the possibly and undoubtedly effective referrals (*n* = 36). As expected, the intervention resulted more effective. The total years saved was 1526 and the cost per year saved was 5.4 US dollars.

## Discussion

This study showed that implementing an effective ambulance referral system to ensure access to EmONC services in a rural setting is highly cost-effective, with a cost per year saved of 24.7 US dollars. This cost is below the 30 US dollars benchmark for the definition of a very attractive intervention and far below the 150 US dollars benchmark for the definition of an attractive intervention [19, 20]. The sensitivity analyses carried on the rate of undoubtedly effective cases, the ambulance costs and the discount rate emphasized the robustness of this conclusion. Moreover, it is worth noting that our results are in line with those observed in a previous similar study carried out in a remote area in Uganda. The cost per year saved in that study was even lower, being 15.8 US dollars [7]. Finally, it has also to be pointed out that our results corroborate the conclusions of other recent studies from different and heterogeneous settings that univocally showed the clinical benefits of an ambulance referral service for EmONC [7–10].

Noteworthy, in our setting, communication and ambulance services were delivered in the context of a comprehensive multi-pronged strategy to address the three delays in seeking appropriate medical care for an obstetric emergency, reaching an appropriate EmONC facility, and receiving adequate care when the facility is reached. We cannot exclude that the attractive economical profile of the ambulance service may be less favourable if the intervention is not concomitantly sustained by a comprehensive approach. Of relevance here is that effectiveness of a referral also depends on timeliness of decision for referral, pre-referral care, en-route stabilizing care (which was not provided in our setting), time taken to arrange referral vehicle, time taken to reach higher facility and promptness with which the case was attended at the higher facility. Regarding comprehensiveness of our intervention, it has also to be underlined that the local framework for supporting maternal and neonatal health was pro-poor oriented. Explicit consideration of how the poor interact with the system and how barriers to access EmONC facility can be overcome was given and, in order to address geographical and financial barriers to access to EmONC services, both ambulance and obstetrical care services were provided free of charge. It is also worth noting that the focus on neonatal and maternal health helps to target those in poverty: in fact not only are death rates higher among the poor compared with the rich, but also the highest poor-rich mortality ratio is observed for complications of pregnancy and delivery [21].

From an economic perspective, it is also worthwhile noting that the ambulance service by itself did not significantly impact on the activity of the hospital. During the three months duration of the study, only 111 ambulance referrals were recorded (8–9 referrals per week). Indirectly, this confirms the previously reported low rate of abuse of the ambulance service [10] and concomitantly excludes a relevant increase in the clinical burden to the hospital. Indeed, the implementation of the service did not lead to an increase in the number of duty personnel. This point is important because, in our study, we decided a priori to include among the costs for the hospital only the material costs needed to provide the assistance, thus assuming that the general burden to the hospital was unremarkable.

Some strengths and limitations of the study should be considered. As for the strengths, all cases were reviewed by health professionals with specific skills on EmONC, thus reducing the risk of misclassifications, while, being a prospective study, erroneous recording of the data was unlikely. Furthermore, important conservative assumptions were made in cost-effectiveness analyses. Concerning costs, an estimated ambulance’s useful life of four years was assumed, as in Uganda [7], while longer useful life was used in other studies carried out in other similar rural areas [22, 23]. Concerning effectiveness, we cannot exclude some misclassification. For instance, some women with obstructive labour may have uterus rupture if not promptly operated: by excluding all women with obstructive labour from the group of undoubtedly effective referrals, we thus presumably under-estimated the effectiveness of the whole program. Furthermore, we exclusively focussed on survival and did not consider quality of life and disability that may also be of relevance. A delayed caesarean section may indeed also impact on quality of life: vesico-vaginal fistulae and child disabilities are overwhelming complications of a delayed caesarean section [24]. Finally, in remote rural, prevention of maternal death may have profound benefits for the entire households [25]. This potential benefit was not included in our analysis. Finally, it is worthwhile noting that, even though the ambulance was meant for obstetrical cases, it was commonly used also for other indications such as, for example, referral of critically ill children with severe anemia who required immediate blood transfusion. The additional benefits of these referrals were herein not considered.

Considering limitations, a possible concern is the accuracy of the classification. In fact, the judgment on effectiveness remains theoretical and there is no way to assess whether referral by other means would have caused the demise of the mother or the foetus This criticism is valid for both clinical and logistics aspects. Regarding the latter, even if initial location and distance from the hospital was considered in the classification of the cases and a personalized judgment was given, this evaluation was subjective. Another debatable point may be the decision to apply the national life expectancy-tables without adjusting for region and pathologies. It may indeed be argued that life expectancy may be lower in the study area and that the application of these tables to all the clinical conditions (such as for instance prematurity or caesarean section) may lead to an overestimate of the benefits. Unfortunately, specific life expectancy-tables for specific areas are not available. Regarding the impact of the indication on life expectancy, it has to be underlined that women and their newborns were discharged only if well and that referrals dying before discharging were considered ineffective. Finally, since the recruitment period was limited to the Winter-Spring seasons, definitive inferences to the whole year cannot be done. All connecting roads in the area were rough and referrals may be more complicated in the rainy season (June to August). The situation of the roads in the area is mostly acceptable even during the rainy season which may explain why we failed to document a significantly lower referral rate during the rainy season in a previous study in the same setting [10].

In the future, the cost-effectiveness profile of the ambulance-based referral system may be improved by ensuring the proper management of uncomplicated cases at Health Center level and addressing inappropriate referrals by training health personnel, improving drug availability and strengthening supervision at peripheral units. An accompanying healthcare professional in the ambulance would be costly, but would improve assistance and would represent a further important step forward. Occasionally, and for the most critical cases, a midwife or a nurse of the hospital usually accompanied the patient. Integration of different means of transport adapted to the local terrain might also increase effectiveness, depending of what is required in terms of distance, geographic terrain, road infrastructure, and weather conditions provided that they do not contrast with local cultural beliefs [6, 26]. Furthermore, taking into consideration that some ambulance costs are mainly fixed and do not significantly increase with the number of referrals, and the rate of institutional deliveries is still low (48%), increasing the number of referrals may further enhance the cost effectiveness profile of the intervention.

## Conclusions

Communication and an ambulance-based referral system for EmONC in remote rural areas appear highly cost-effective. However, the mere implementation of this single intervention may be insufficient if not included in the context of a comprehensive multi-pronged strategy aimed at ensuring continuity of care and at strengthening referral.

## Abbreviations

EmONC: Emergency Obstetric and Neonatal Care
GDP: Gross Domestic Product
US: United States
